# Evaluation and Treatment of Female Sexual Pain: A Clinical Review

**DOI:** 10.7759/cureus.2379

**Published:** 2018-03-27

**Authors:** James Sorensen, Katherine E Bautista, Georgine Lamvu, Jessica Feranec

**Affiliations:** 1 University of Central Florida Ucf Com/hca Gme Consortium Obstetrics and Gynecology Residency Program, UCF/ Orlando Va Medical Center; 2 Women Center, Department of Veteran Affairs; 3 Division of Surgery, Gynecology Section Orlando Va Medical Center, University of Central Florida College of Medicine , Orlando, USA; 4 Division of Surgery, Gynecology Section Orlando Va Medical Center, University of Central Florida College of Medicine, Orlando, USA

**Keywords:** dyspareunia, vulvodynia, sexual pain, pelvic pain, gynecology

## Abstract

Dyspareunia and vulvodynia are genital pain disorders that have devastating effects on women’s quality of life. These disorders occur with high prevalence and place a significant financial burden on women and the health care system. Many women do not report genital pain, and most providers do not inquire about this type of pain. As a result, women also experience social isolation. Numerous treatments are thought to improve quality of life and decrease pain; however, more studies are needed. This review aims to provide an overview of clinical evaluation methods and to summarize treatment options for women suffering from dyspareunia and vulvodynia.

## Introduction and background

The medical term for painful intercourse is dyspareunia. This definition includes recurrent or persistent discomfort that happens before, during, or after intercourse. Dyspareunia is a complex disorder that can be further classified as superficial or deep, and primary or secondary [1-2]. Superficial dyspareunia is pain localized to the vulva or vaginal entrance, and deep dyspareunia is pain perceived inside the vagina or lower pelvis, which is often associated with deep penetration [1-2]. Primary dyspareunia occurs at initial intercourse, and secondary dyspareunia occurs after some time of pain-free intercourse.

Painful intercourse is sometimes further characterized as vulvodynia. Vulvodynia is a chronic pain that is defined as genital pain with no known etiology that lasts more than three months and may or may not be associated with sexual intercourse [3]. The International Society for the Study of Vulvovaginal Diseases (ISSVD), the International Society for the Study of Women’s Sexual Health (ISSWSH), and the International Pelvic Pain Society (IPPS) further describe vulvodynia by the site of pain (localized, generalized, or mixed); if it is provoked, spontaneous, or mixed; or if the pain is intermittent, persistent, constant, immediate, or delayed [3]. Localized vulvodynia refers to pain limited to the vulvar vestibule around the hymeneal ring at the entrance to the vagina, and generalized vulvodynia is defined as pain affecting the entire vulvar region [2]. Vulvodynia has no clear etiology. However, ISSVD, ISSWSH, and IPPS list the following potential associated factors: other pain syndromes, genetics, hormonal factors, inflammation, musculoskeletal or neurologic mechanisms, psychosocial factors, and structural defects [3].

Dyspareunia and vulvodynia are often used interchangeably; however, it is important to appreciate that the terms have different meanings. Dyspareunia is a descriptive term for the symptom of pelvic or vaginal pain associated with intercourse (i.e., it describes pain that always occurs with provoking touch such as intercourse). However, vulvodynia may occur with or without provocation (i.e., spontaneously). Dyspareunia can occur at the entrance of the vagina, deep in the vaginal canal, or in the pelvis. Vulvodynia is localized to the vulva and vaginal introitus. Lastly, whereas dyspareunia may be acute or chronic, vulvodynia is a term used specifically for the classification of chronic pain (i.e., pain lasting longer than three months). Both terms can be used to describe pain that coexists with other comorbidities such as endometriosis, interstitial cystitis, pelvic floor myalgias, and vulvar dermatoses. 

## Review

Prevalence and burden of dyspareunia and vulvodynia

The prevalence of dyspareunia and vulvodynia varies by how they are defined and by geographic location. For example, the prevalence of dyspareunia in the United States is approximately 10% to 20%, with the leading causes varying by age group [4]. The World Health Organization reported a global prevalence of painful intercourse ranging between 8% and 21.1% in 2006, which varied by country. In a 2016 systematic review in Brazil, the prevalence of dyspareunia ranged from 1.2% to 56.1%, which differs from Puerto Rico’s prevalence rate of 17% to 21% [5-6].

In a comprehensive review, it was shown that vulvodynia has an estimated prevalence range of 10% to 28% in reproductive-aged women in the general population [7]. Approximately 8% of women aged 18 to 40 years old have reported a history of vulvodynia that limited or prevented intercourse [8]. Other studies have also found a higher prevalence of vulvar pain in Hispanic women compared to white women [8-10].

Women who suffer from chronic genital pain find it difficult to seek help, treatment, or support, and, as a result, they often experience significant social isolation [7]. In a questionnaire on sexual experience and dyspareunia, 48% of women who suffer from dyspareunia reported sexual dysfunction and decreased sexual frequency [11]. Women who have comorbidities such as endometriosis, fibroids, or vaginitis related to dyspareunia also have lower sexual function that causes relationship distress with their partners and decreases their quality of life [12]. Studies have also shown a significant correlation between sexual pain and psychiatric comorbidities such as depression and anxiety [12].

In addition to the burden that dealing with pelvic pain adds to women's lives, a significant share of health care dollars is spent on treating pelvic pain. A four-year cross-sectional Canadian study revealed that more than $100.5 million (with an average cost of $25 million per year) was spent on treating chronic pelvic pain disorders, with dyspareunia accounting for 6.6% of the cost [13]. Based on the reported vulvodynia prevalence of 3% to 7% in the US, the national burden cost of vulvodynia ranges from $31 to $72 billion annually [14].

Etiology and risk factors of dyspareunia and vulvodynia

Dyspareunia is believed to be a specific pain disorder with interdependent psychological and biological etiologies. Like vulvodynia, superficial dyspareunia can be associated with vaginitis, dermatosis, and vulvovaginitis [1]. In contrast, deep dyspareunia can result from visceral disorders such as interstitial cystitis pelvic inflammatory disease, endometriosis, adhesions, pelvic congestion, and fibroids [1,15]. Pain syndromes can potentially overlap and be associated with dyspareunia and vulvodynia, including irritable bowel syndrome, fibromyalgia, and musculoskeletal dysfunction [1,3,7].

Other conditions that may contribute to the development of dyspareunia include poor vaginal lubrication, vaginal atrophy, and childbirth [1,16]. Childbirth is a risk factor for developing pelvic pain and/or dyspareunia during and potentially beyond the postpartum period [17]. A cross-sectional study on the effects of childbirth on sexual health reported that an estimated 17% to 36% of women reported dyspareunia at six months postpartum, yet only 15% of women who had postnatal dyspareunia discussed it with a health provider [16,18].

Associated comorbidities that may cause vulvodynia are vulvar/vaginal infections, inflammation, neoplasms, trauma, iatrogenic or hormonal deficiencies, neuropathic pain or pelvic floor muscle dysfunction, structural defects, and psychosocial factors [3,19-20].

Clinical evaluation

Patient History

Women suffering from dyspareunia may struggle to find support and acknowledgment that their pain is “real.” Many women report being dismissed and invalidated [21]. Thus, the first step in evaluating the patient should include validation of the patient’s pain and establishing rapport and trust between the patient and provider. The next step in the evaluation should include obtaining a detailed history that reviews the following: 1) pain characteristics (location, duration, exacerbating factors); 2) associated symptoms such as bowel, bladder, or musculoskeletal symptoms; 3) sexual behavior and sexuality; 4) psychological history; 5) comorbid medical problems; 6) previous treatments; and 7) physical or sexual abuse (Table 1) [20].

**Table 1:** Important elements to discuss during clinical evaluation of female sexual pain

Medical History Questions			
PAIN CHARACTERISTICS		Timing, duration, quality, location, provoked, or unprovoked	
MUSCULOSKELETAL HISTORY		Pelvic floor surgery, trauma, obstetrics	
BOWEL AND BLADDER HISTORY		Constipation, diarrhea, urgency, frequency	
SEXUAL HISTORY		Frequency, desire, arousal, satisfaction, relationship	
PSYCHOLOGICAL HISTORY		Mood disorder, anxiety, depression	
HISTORY OF ABUSE		Sexual, physical, neglect	

Providers should obtain a general medical and surgical history before progressing to a gynecologic, obstetric, and sexual history [19,20]. The overall goal of the medical interview should be to validate the patient’s symptoms while gathering relevant information, excluding other diagnoses and educating and reassuring the patient [19-21].

When quantifying the pain, validated self-report questionnaires such as the Female Sexual Function Index, the McGill Pain Questionnaire, or the Patient Reported Outcomes Measurement Information System (PROMIS) vulvar discomfort scale may be more helpful rather than asking a patient to rate her pain on one to 10 [20]. Specific questionnaires allow patients to provide clinicians a better understanding of the quality and intensity of their pain and the impact it has on their lives. In addition, questionnaires are efficient and allow providers to collect a large amount of information in the limited clinical time available for face-to-face interaction. 

Physical Exam

Patients want information about the examination process before, during, and after the evaluation is performed. Therefore, it is helpful to begin the physical inspection by first educating the patient about the examination and her anatomy while explaining what information has been obtained from each step of the assessment [20,22-23].

Physical examination of patients with genital pain should include an external musculoskeletal evaluation, followed by external visual and sensory examination, as well as internal single digit palpation of the pelvic floor muscles. If tolerated by the patient, the provider may proceed to a bimanual examination and a speculum exam. It is important to recognize the possible discomfort and anxiety associated with assessments of the pelvis, particularly in patients with pain. A common strategy used to minimize anxiety and discomfort is the interactive educational pelvic examination process, which includes 1) explanations to the patient while performing the assessment; 2) describing the specific actions during each step; 3) using a mirror to enable the patient to visualize her anatomy and the examination [20,24]. This allows the clinician to thoroughly evaluate the patient’s pain, exclude diagnoses, educate the patient regarding normal anatomy and sexual function, and reassure the patient when no pathology is uncovered [1,19].

The external musculoskeletal examination includes a complete lower back, abdomen, and pelvic inspection. It begins by observing any asymmetry or pain in the patient’s gait and her posture in the standing and sitting positions [1,25]. Next, the abdominal, gluteal, back, and lower extremity muscles are palpated to identify areas of tension and/or pain [20]. Last, an assessment of muscle strength, range of motion, sensation, and reflexes should be performed [25].

**Figure 1:**
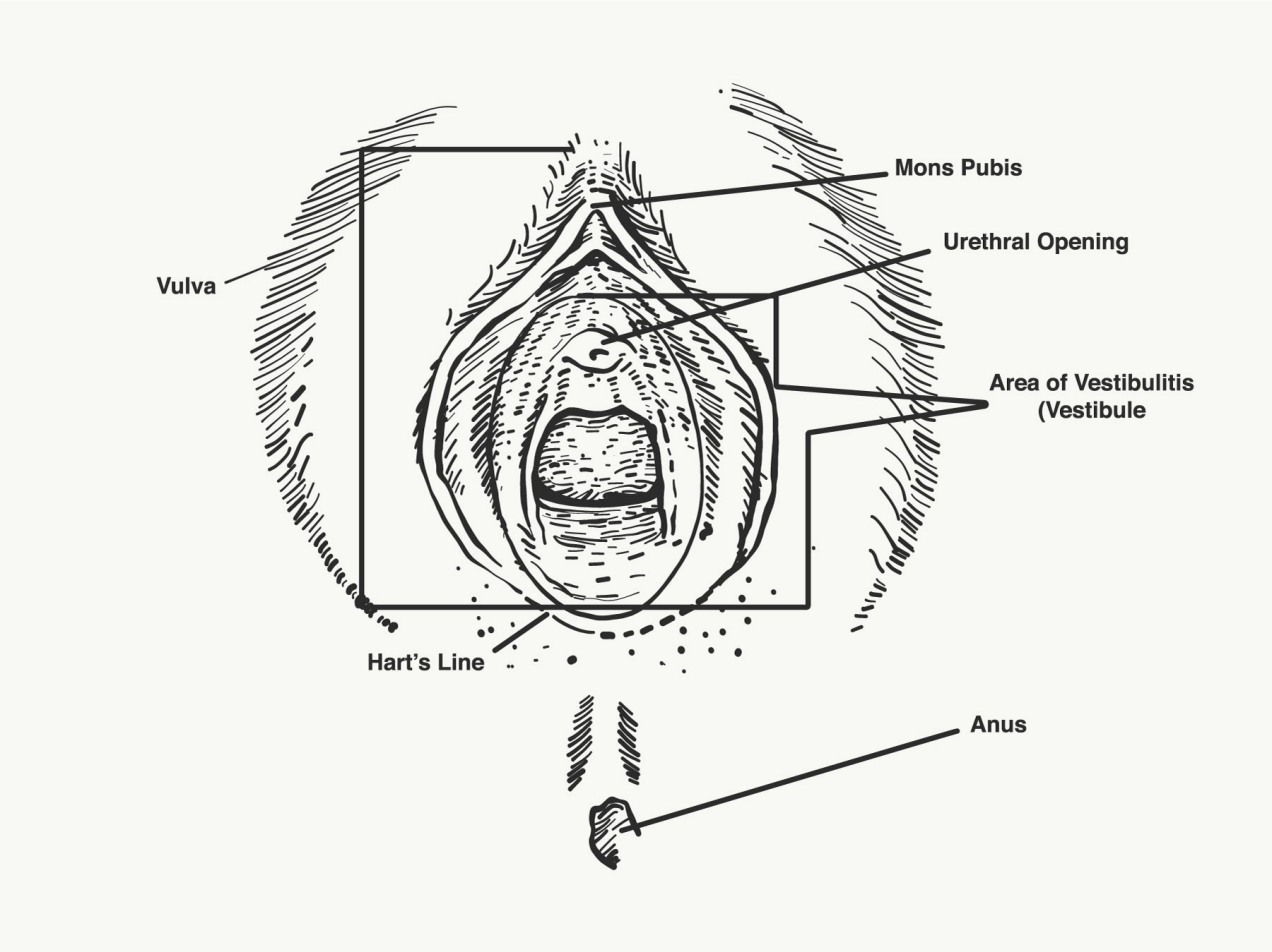
Vulvar anatomy

**Figure 2:**
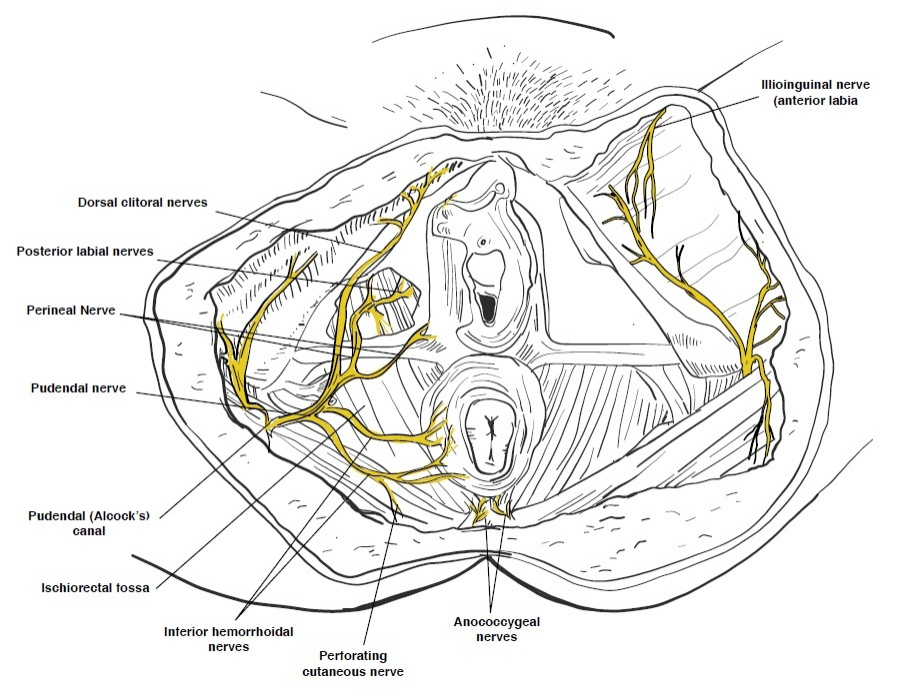
Vaginal sensory innervation

**Figure 3:**
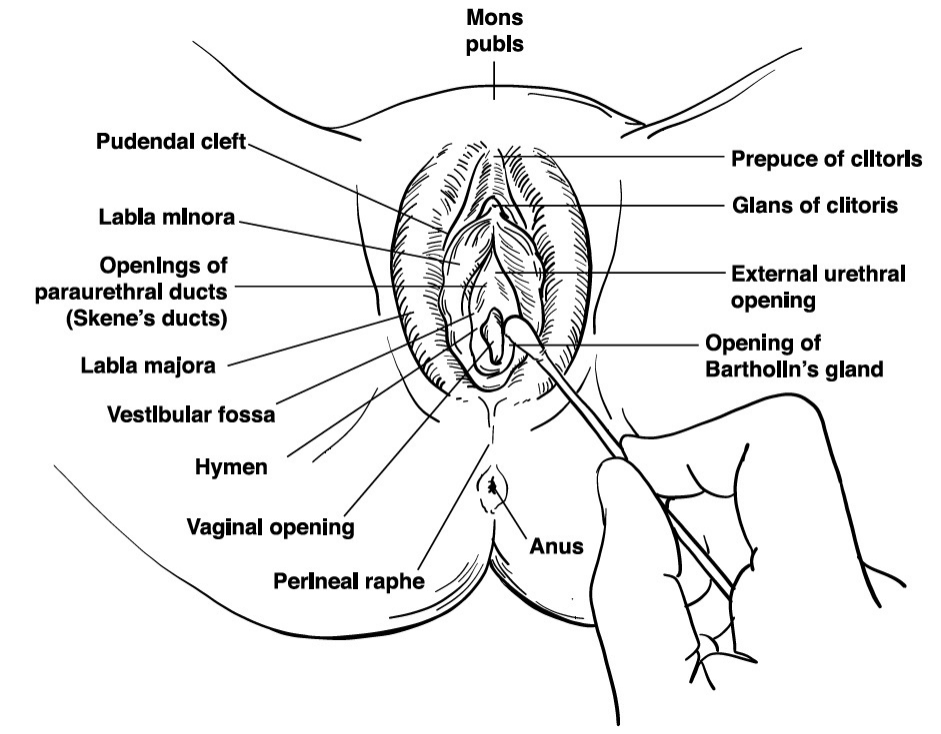
Vaginal cotton swab examination

The vulvar examination is performed systematically by inspecting the external genitalia, perineum, perianal areas, and mons pubis, and assessing for the presence of infection, trauma, atrophy, fissure, and dermatosis (Figures 1-2) [1]. The standard test for diagnosis of vulvodynia is the cotton swab test. This test can help determine the location of pain as well as distinguish between mechanical allodynia and hyperalgesia [1,20]. Allodynia is a term used to describe a painful response to a non-painful stimulation, such as light touch with a cotton swab. Hyperalgesia is an excessively painful response to a painful stimulus. The examiner can use the cotton-tipped applicator technique to conduct a sensory exam of the vulva and the six anatomical sites on the vestibule. The clock face is used as a reference when describing the location of the vulva and pelvic structures. The 12 o’clock and six o’clock correspond to the anterior and posterior midline or pubic symphysis and anus, respectively (Figure 3) [1,25-26]. The presence of allodynia or hyperalgesia on the vestibule is abnormal and suspicious for neuropathy.

**Figure 4:**
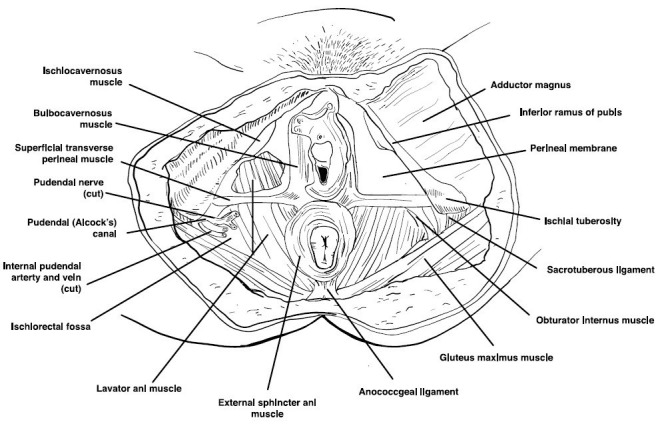
Pelvic floor muscles

The internal musculoskeletal and vaginal single-digit exam is the most reliable method for evaluating pelvic muscle tenderness [25]. Using the index finger, the examiner can palpate the lateral, anterior, and posterior walls of the vagina, the urethra, and pelvic floor muscles (levator ani, coccygeus, piriformis, and obturator internus). The purpose is to access the specific areas for tone, tenderness, or involuntary spasms of the muscles of the introitus and pelvic floor (Figure 4) [1,25]. Tenderness during minimal or moderate palpation is considered abnormal; pelvic and vaginal structures can tolerate approximately 2 kg of pressure without pain. The patient is then asked to squeeze or contract around the single digit to assess their muscle strength. An effort should also be made to identify any scars from previous surgeries, episiotomy, or trauma.

If the patient can tolerate the single digit muscular exam, a bimanual exam can be performed to evaluate the uterus and adnexa. The purpose is to assess the uterus, cul-de-sac, and adnexa regions for any masses or tenderness [1,19]. If the patient tolerates this portion of the exam, then the provider can proceed to an internal examination using a small-sized Grave’s or Pederson speculum. All efforts should be made to insert the speculum slowly to allow accommodation of the speculum and to avoid touching the urethra or vulvar vestibule which can elicit pain [1]. During the speculum exam, the internal vaginal tissue, cervix, and vaginal secretions are examined. At this time, cultures or biopsies can be collected to rule out infections, dermatoses, or abnormal cellular dysplasia that can cause dyspareunia or vulvodynia.

Treatment

Current treatment recommendations for sexual pain, dyspareunia, and vulvodynia vary. The selected treatment should be specific if a cause is identified (e.g., vaginal infection, musculoskeletal, endometriosis). Because vulvodynia is chronic genital pain, medical treatment for vulvodynia may be more challenging. Treatments should be individualized, and a multimodal treatment approach to address all aspects of pain (i.e., physical, emotional, and behavioral) is recommended.

Both dyspareunia and vulvodynia treatment plans, if appropriate, should start with conservative medical non-invasive approaches. It will rarely be necessary to escalate to surgical interventions. This may involve a team approach with gynecology, physical therapy, pain management, sexual therapy, and mental health professionals who specialize in chronic pain [27].

Education

The first step in the treatment process is acknowledging and validating that the patient has pain [1]. Patients should be instructed that resolution of their pain might be a long process or that the pain may not completely resolve. Providers should focus on educating patients about pelvic anatomy, physiology, and lifestyle modification. It is particularly important to review appropriate vulvar care to minimize vulvar irritation such as wearing 100% cotton underwear, applying preservative-free and alcohol-free emollients or lubricants during intercourse, and avoiding irritants (e.g., perfumes, dyes, shampoos, detergents), harsh soaps, douching, and hair drying the vulvar area [1,26].

Patients should be informed of their treatment options, so that they feel empowered to make an informed medical decision. Medical therapies for dyspareunia and vulvodynia include topical anesthetics, oral tricyclic antidepressants, oral or topical hormonal treatments, oral anti-inflammatory agents, Botox and trigger point injections, physical therapy, cognitive behavioral therapy, and other types of brain-based therapies, or surgery.

Local Anesthetics

Sensitization of peripheral vestibular nerves has been suggested as a possible mechanism of pain in vulvodynia [20]. Thus, topical anesthetics, such as lidocaine, can be used to relieve pain during intercourse [1]. These medications are also useful for short-term therapy and in combination with other therapies (e.g., physical therapy and botulinum toxin). Local anesthetics are theorized to desensitize peripheral vulvar and vaginal nerves and achieve pain relief. Typically, topical 5% lidocaine is used once or twice daily with reevaluation after six to eight weeks of use [20].

Hormonal Treatment

Vulvovaginal atrophy caused by decreased levels of estrogen is a common problem in aging women. In patients who present with the main symptoms of atrophy, dryness, and dyspareunia, the first line of therapy consists of topical estrogen to restore normal vaginal pH levels and thicken and increase cell numbers leading to the revascularization of the epithelium [1,28]. Low-dose conjugated estrogens available in the forms of vaginal inserts (e.g., cream, tablet, and ring) can be applied periodically from a few times a week to every three months. Topical estrogen should be considered to avoid the systemic effect of oral estrogen. However, patients using estrogen supplementation in any form should be followed clinically [28], and estrogen supplementation is contraindicated in patients with certain comorbidities such as breast cancer and uncontrolled cardiovascular disease. Additionally, topical vaginal estrogen therapy may take up to four weeks before patients notice an effect.

Anti-Inflammatory Agents

Tissue levels of interleukin-B, an inflammatory mediator cytokine, have been reported to be higher in the hymenal region of the vestibule of women with vulvodynia [20,29]. Injectable anti-inflammatory agents such as corticosteroids, interferons, and mast cell stabilizers have been used to treat vulvodynia with some improvement. However, randomized controlled trials to recommend them as a first-line treatment are lacking [20].

Botulinum Type A

Injection of botulinum toxin A into the pelvic floor muscles has been shown in some studies to decrease dyspareunia and vulvodynia caused by pelvic floor myalgia and contracture [30-31]. Botulinum toxin A is hypothesized to inhibit nociceptors leading to a decrease in peripheral and central sensitization associated with vulvodynia [20]. A long-term assessment of the effectiveness of botulinum injections after 24 months revealed that patients could have sexual intercourse and had improved quality of life [31]. It is not recommended as a first-line therapy as further clinical trials of botulinum type A are needed, but it is used as an adjunct to other therapies [20].

Systemic Medications

Tricyclic antidepressants and anticonvulsants have been shown to improve pain symptoms in patients with vulvodynia [1,26]. Tricyclic antidepressants such as amitriptyline are known to reduce peripheral nerve sensitization and have been used in the management of neuropathic pain [27]. It can take up to three weeks to achieve pain control [27]. Although patients have reported symptomatic relief from vulvodynia with tricyclic pharmacotherapy, additional research is required to identify the characteristics that would predict the appropriate patients for this therapy [20].

Physical Therapy and Behavioral Therapy

Pelvic floor physical therapy is an important adjunct to most treatments for dyspareunia and vulvodynia [1,32]. It allows the pelvic floor muscles to relax and retrains pain receptors. In a systematic literature review, physical therapy modalities such as biofeedback, dilators, electrical stimulation, education, physical therapy, and multidisciplinary treatments were effective in decreasing pain during intercourse and improving sexual function [33].

In conjunction with other therapies, cognitive behavioral therapy has been shown to be effective in reducing the anxiety and fear related to dyspareunia [34]. It is the most commonly used and studied behavioral intervention [20]. Cognitive behavioral therapy focuses on patterns of thinking and helps identify behaviors associated with negative thoughts and feelings. It is also an effective non-invasive and safe therapeutic option and is highly recommended in the management of vulvodynia [20].

Surgical Therapy

Surgical treatment is performed as a last resort when all conservative and medical management options have failed or when surgery is indicated to determine and/or treat pelvic adhesions, endometriosis, or pelvic organ prolapse. The surgical options are specific to the disorder, but most commonly include vulvar vestibulectomy, lysis of pelvic adhesions, or excision of endometriosis [1,20,35]. Thorough counseling is necessary prior to pursuing surgical treatment. Patients must understand that surgery may improve their pain, and their pain may sometimes return or worsen.

Vestibulectomy can be an effective treatment only for localized, provoked vestibulodynia. Studies have demonstrated that surgical management of provoked vestibulodynia can lead to significant pain relief in 60% to 90% of patients [20,36]. Generally, surgical techniques include a complete vulvar vestibulectomy, which involves excision of the mucosa of the entire vulvar vestibule and the mucosa adjacent to the urethra, or a modified vestibulectomy, which limits the excision of mucosa to the posterior vestibule [37].

## Conclusions

Despite the prevalence and impact of dyspareunia, many women do not seek care. Women with dyspareunia often suffer in silence and feel that their pain has not been assessed or validated by providers. Dyspareunia and vulvodynia can be challenging to diagnose and may require a multidisciplinary approach; therefore, a comprehensive and systematic exam is required to understand the specific causes of genital pain. Treatments often involve multimodal approaches that include education, medication, cognitive behavioral therapy, physical therapy, and possibly surgery.
